# Towards patient-centred care in Ghana: health system responsiveness, self-rated health and experiential quality in a nationally representative survey

**DOI:** 10.1136/bmjoq-2019-000886

**Published:** 2020-05-12

**Authors:** Hannah L Ratcliffe, Griffith Bell, Koku Awoonor-Williams, Asaf Bitton, June-Ho Kim, Stuart Lipstiz, Erlyn Macarayan, Anthony Ofosu, Easmon Otupiri, Dan Schwarz, Lisa R Hirschhorn

**Affiliations:** 1 Ariadne Labs, Brigham and Women’s Hospital & Harvard T.H. Chan School of Public Health, Boston, Massachusetts, United States; 2 Division of Policy, Planning, Monitoring and Evaluation, Ghana Health Service, Accra, Greater Accra, Ghana; 3 Division of General Internal Medicine and Primary Care, Department of Medicine, Brigham and Women's Hospital, Boston, Massachusetts, USA; 4 Harvard Global Health Institute, Harvard University T.H. Chan School of Public Health, Cambridge, Massachusetts, USA; 5 Ghana Health Service, Accra, Greater Accra, Ghana; 6 Department of Population, Family and Reproductive Health, Kwame Nkrumah University of Science and Technology, Kumasi, Ashanti, Ghana; 7 Division of Global Health Equity, Department of Medicine, Brigham and Women's Hospital, Boston, Massachusetts, USA; 8 Department of Medical Social Science, Northwestern University Feinberg School of Medicine, Chicago, Illinois, USA

**Keywords:** global health, patient-centred care, patient satisfaction, quality measurement

## Abstract

**Introduction:**

Person-centredness, including patient experience and satisfaction, is a foundational element of quality of care. Evidence indicates that poor experience and satisfaction are drivers of underutilisation of healthcare services, which in turn is a major driver of avoidable mortality. However, there is limited information about patient experience of care at the population level, particularly in low-income and middle-income countries.

**Methods:**

A multistage cluster sample design was used to obtain a nationally representative sample of women of reproductive age in Ghana. Women were interviewed in their homes regarding their demographic characteristics, recent care-seeking characteristics, satisfaction with care, patient-reported outcomes, and—using questions from the World Health Survey Responsiveness Module—the seven domains of responsiveness of outpatient care to assess patient experience. Using Poisson regression with log link, we assessed the relationship between responsiveness and satisfaction, as well as patient-reported outcomes.

**Results:**

Women who reported more responsive care were more likely to be more educated, have good access to care and have received care at a private facility. Controlling for respondent and visit characteristics, women who reported the highest responsiveness levels were significantly more likely to report that care was excellent at meeting their needs (prevalence ratio (PR)=13.0), excellent quality of care (PR=20.8), being very likely to recommend the facility to others (PR=1.4), excellent self-rated health (PR=4.0) and excellent self-rated mental health (PR=5.1) as women who reported the lowest responsiveness levels.

**Discussion:**

These findings support the emerging global consensus that responsiveness and patient experience of care are not luxuries but essential components of high-performing health systems, and highlight the need for more nuanced and systematic measurement of these areas to inform priority setting and improvement efforts.

## Introduction

The year 2018 saw the release of three major reports on improving quality of care. These reports, from the Lancet Global Health Commission on High-Quality Health Systems,^1^ Institute of Medicine,^2^ and World Bank Group, World Health Organization (WHO) and the Organisation for Economic Co-Operation and Development,^3^ point towards an increased focus on measuring and improving quality of care, particularly in low-income and middle-income countries (LMIC). Although the three reports vary in their areas of focus and recommendations, the root of the argument in each is that the global goal of universal health coverage cannot meaningfully be achieved without focusing on improving the quality, not just coverage, of healthcare services.

**Figure 1:**
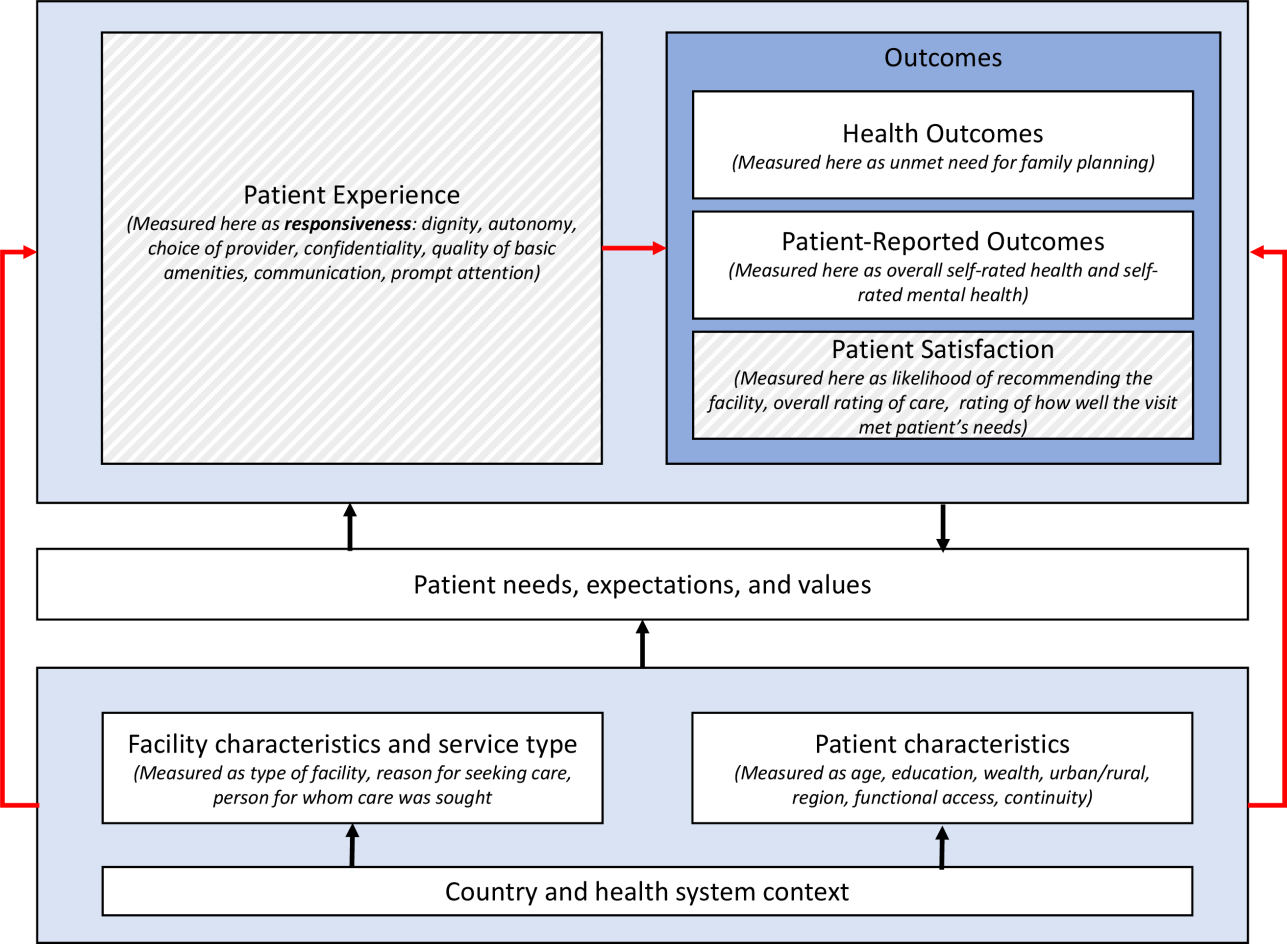
The hypothesised relationship between respondent characteristics, visit characteristics and service type, and patient expectations with patient experience (measured as responsiveness) and outcomes, including patient satisfaction and patient-reported outcomes. Red lines indicate the relationships assessed in this study. Dashed fill indicates components of ‘person-centredness’. Figure modified from Larson *et al*.^4^

The Lancet Global Health Commission on High-Quality Health Systems defines four foundational values of high-quality health systems: person-centred, equitable, resilient and efficient.^1^ Person-centredness is both intrinsically and instrumentally important for ensuring quality,^4^ intrinsically because all people have the right to receive dignified and respectful care that is responsive to their needs^5^ and instrumentally because person-centredness has been associated with improved healthcare utilisation and better health outcomes.^6^ Larson and colleagues^4^ propose two categories of measurement of person-centredness: (1) patient experience of care, a process measure; and (2) patient satisfaction, defined as an outcome measure of how well provided care meets patient needs and expectations (figure 1). Both patient experience and patient satisfaction are important metrics for holding health systems accountable for improving quality of care and being responsive to user expectations.^4^

One of the first frameworks for measuring patient experience of care was the WHO’s conceptualisation of responsiveness of care.^5^ According to their definition, responsiveness is comprised eight components: dignity, autonomy, confidentiality, clear communication, choice of care provider, prompt attention, quality of basic amenities and access to social support networks during inpatient care.^5 7^ The WHO identifies responsiveness of care as an intrinsic goal of health systems, on par with improving and maintaining health and fair financing and financial risk protection.^8^

Despite the theoretical importance placed on responsiveness, however, in the nearly 20 years since the WHO first defined the concept, few studies have explored this essential function of health systems, particularly at the population level in LMIC.^1 9^ The World Health Report 2000 examined responsiveness across countries, but relied on a relatively small number of key informant interviews and thus did not generate findings that were generalisable at the population level.^10^ In 2008, Valentine *et al*^7^ conducted a cross-country study to determine which components of responsiveness were most important to people, but did not measure overall levels of responsiveness. Multiple other studies have examined responsiveness among specific populations^11–14^ or looked at the correlation of contextual factors such as healthcare expenditure on average responsiveness at the country level.^15 16^ More recently, Geldsetzer *et al*^9^ determined the prevalence of non-responsive care among older adults in six middle-income countries.^9^ Their study was one of the first to look at individual reports of responsiveness of care at the population level and found high rates of non-responsive care and significant inequities across and within countries, highlighting the need for more focus on how to measure and improve this critical element of quality of care.

In addition to a general lack of information about how responsiveness varies within and across populations, few studies have examined the relationship at the individual level between responsiveness and other important health system outcomes, including patient satisfaction and patient-reported outcomes.^17^ In this paper, we use data from a nationally representative sample of women of reproductive age (15–49 years) in Ghana, where the Geldsetzer *et al*’s study found comparatively low levels of responsiveness among elderly populations.^9^ We explore the predictors of responsiveness and estimate the associations between responsiveness and important health system outcomes such as patient satisfaction, overall self-rated health, self-rated mental health and unmet need for family planning.

## Methods

### Survey design and fielding

The conduct and reporting of this study are aligned with the Strengthening the Reporting of Observational Studies in Epidemiology guidelines (online supplementary file 1). We used data collected from a nationally representative household survey in Ghana conducted in 2017 by the Performance Monitoring and Accountability 2020 programme (PMA2020). PMA2020 is funded by the Bill & Melinda Gates Foundation to use mobile technology to provide rapid-turnaround data on progress towards key health and development indicators.^18^ Detailed descriptions of the survey and methodology have been published elsewhere.^18 19^ Briefly, PMA2020 collected data on a nationally representative sample of women of reproductive age (ages 15–49 years). The survey employed a multistage cluster sample design to calculate a probability sample of households and eligible women in order to estimate the country-wide modern contraceptive prevalence rate. The sample was selected from a master sampling frame of enumeration areas—each consisting of approximately 200 households—provided by the Ghana Statistical Service using a probability proportional to size framework stratified by urban and rural areas. Within each enumeration area, 42 households were randomly selected to complete a household survey, and all women of reproductive age in each sampled household were asked to complete an individual survey. Study staff from the local enumeration areas were trained to administer the surveys. The household survey included a household roster and questions regarding household assets and wealth. The individual survey included two components: (1) the standard core PMA2020 survey including sociodemographic information and reproductive health^20^; and (2) a novel survey component, designed by the study team, which asked respondents who had sought care in the last 6 months about their experience accessing and receiving healthcare services, including their reasons for seeking care and self-rated overall health and mental health.

10.1136/bmjoq-2019-000886.supp1 Supplementary data

### Variables

#### Respondent and visit characteristics

Our survey collected information on both respondent and visit characteristics.

Respondent characteristics were as follows:

- Age category.
- Highest level of education.
- Wealth (as determined by a wealth index created by PMA2020 based on household characteristics, determined based on possession of livestock, durable goods, and features of the dwelling unit, including wall, floor and roof materials, water sources, and sanitation facilities).
- Urban/rural residence.
- Region of residence.
- Functional access to care, defined as participants’ perceived ease of receiving care tomorrow, if needed, measured on a 5-point Likert scale.
- How frequently respondents reported seeing the same provider at each visit, measured on a 5-point Likert scale.

Visit characteristics, for the most recent visit in the last 6 months, were as follows:

- Type of facility visited.
- Reason for seeking care, classified as acute, chronic or preventive.
- Whether the care sought was for the respondent or others.

#### Responsiveness

Responsiveness was assessed using the framework and survey questions established by the WHO’s World Health Survey Responsiveness Module.^5^ Respondents were asked to assess their last care-seeking experience on each of the seven domains of responsiveness relevant to outpatient care. The following were the dimensions, along with the specific survey questions used to assess them:

- Dignity (How would you rate the level of respect the provider showed you?)
- Autonomy (How would you rate your experience of being involved in making decisions for your treatment?)
- Choice of provider (How would you rate the ease with which you could see a healthcare provider you were happy with?)
- Confidentiality (How would you rate the way that health services ensured that you could talk privately to providers?)
- Quality of basic amenities/surroundings/environment (How would you rate the cleanliness of the facility?)
- Communication (How would you rate the provider’s availability to explain things in a way that you could understand?)
- Prompt attention (How would you rate the length of wait time at the facility before you were seen?)

Each question was assessed using a Likert scale rating (poor, fair, good, very good, excellent) and responses were recoded on a scale of 1 (poor) to 5 (excellent). A responsiveness index was created using a scaled mean of the seven scores. Once compiled, the index was partitioned into five quintiles for ease of interpretation.

#### Patient satisfaction and patient-reported outcome measures

Patient satisfaction and patient-reported outcome measures were measured using 5-point Likert scales and were chosen a priori based on expert recommendation and availability of data through the PMA2020 platform. These included the following:

- Patient satisfaction with most recent care-seeking experience.
  - Likelihood of recommending the facility to others.
  - Overall rating of care received at the facility.
  - Rating of how well care at the facility met the respondent’s health needs.
- Patient-reported outcomes.
  - Overall self-rated health.
  - Self-rated mental health.
- Unmet need for family planning.

### Statistical analysis

We excluded 1.7% (34 of 1946) women who did not answer all survey questions in the fully adjusted analysis. Distributions of variables between the highest and lowest responsiveness quintiles were compared using simple descriptive statistics. Poisson regression with a log link and robust standard errors (SE) were used to estimate prevalence ratios (PR) and 95% confidence intervals (CI) for the relationship between the quintile of responsiveness index and the highest rating of each of the main outcomes. The PRs are obtained by exponentiating the Poisson regression coefficients. All analyses accounted for the complex survey design by incorporating survey weights, strata and cluster variables in order to accurately reflect the nationally representative survey population.

To assess the relationship between responsiveness and outcomes, we first fit crude models unadjusted for any other variables. We then fit a series of staged models adjusting for respondent characteristics and visit characteristics. Adjustment variables were chosen a priori and informed by scientific literature review based on whether they might impact both responsiveness and study outcomes. P values were considered significant at the alpha level <0.05. Analyses were performed using Stata V.15.1.

### Patient and public involvement

This research was done without patient involvement; however, the findings have been shared at a national dissemination event involving government officials and civil society organisations to ensure findings can inform future policy.^21^

All study participants provided informed, written consent.

## Results

### Respondent and visit characteristics

Of the 4322 participants surveyed, 2018 (47%) reported having visited a healthcare facility within the last 6 months. After accounting for the complex survey design, the total weighted study sample was 1946. The characteristics of women included in the sample are presented in table 1. Demographic characteristics of the full sample can be found in online supplementary table 1.

10.1136/bmjoq-2019-000886.supp2 Supplementary data

**Table 1:** Demographics and visit characteristics by responsiveness index quintile

	Quintile of responsiveness index (RI)					
	Lowest RI quintile (n=549)		Highest RI quintile (n=404)		Total (N=1946)	
	n	%	n	%	n	%
Respondent characteristics						
Age (n=1946)						
15–24	163	29.7	103	25.4	550	28.3
25–34	215	39.2	153	37.7	738	37.9
35–49	171	31.1	149	36.9	658	33.8
Primary or lower schooling (n=1946)	225	40.9	131	32.5	677	34.8
Income (n=1946)						
Bottom two wealth quintiles	234	42.6	177	43.7	774	39.8
Neighbourhood (n=1946)						
Urban	266	48.4	190	47.0	971	49.9
Rural	283	51.6	214	53.0	975	50.1
Region of residence (n=1946)						
Ashanti	58	10.6	116	28.7	400	20.6
Brong Ahafo	42	7.6	29	7.1	110	5.7
Central	17	3.2	101	25.1	178	9.1
Eastern	112	20.3	20	4.9	229	11.8
Greater Accra	94	17.1	48	11.8	291	14.9
Northern	34	6.2	48	11.9	202	10.4
Upper East	66	11.9	3	0.8	133	6.8
Upper West	39	7.1	0	0	72	3.7
Volta	33	6.0	25	6.2	123	6.3
Western	55	10.0	14	3.5	207	10.7
Very easy to get care if needed tomorrow (n=1941)	304	55.4	344	86.6	1364	71.0
Always see same provider (n=1943)	117	21.3	100	24.7	438	22.6
Visit characteristics						
Facility type (n=1912)						
Community-based health planning and services	58	10.6	61	15.4	210	11.0
Government hospital/polyclinic	242	44.3	163	41.0	777	40.6
Government health centre	161	29.6	60	15.1	464	24.2
Private hospital/clinic	65	11.9	89	22.4	353	18.5
Other	19	3.6	24	6.0	108	5.7
Reason for seeking care* (n=1946)						
Preventive	137	24.9	129	31.8	533	27.4
Chronic	21	3.9	5	1.3	66	3.4
Acute	391	71.2	270	66.9	1347	69.2
Seeking care for self only (n=1946)	290	52.9	211	52.3	1054	54.2
Seeking care for others only (n=1946)	174	31.7	117	28.9	599	30.8
Seeking care for self and others (n=1946)	85	15.4	76	18.8	293	15.1

Counts and percentages weighted to account for survey sampling design (unweighted n=2018).

*Acute: any fever, sick, snake bite, injury, worried about a new symptom, community health worker instructed to go, any eye issue, abdominal pain or respiratory problem. Chronic: nothing classified as acute, and blood pressure, HIV or diabetes. Preventive: nothing classified as acute or chronic, and check-up, family planning, maternal care, vaccination or other.

There was some notable variation in respondent and visit characteristics between women in the highest responsiveness category and those in the lowest. Women in the highest responsiveness category had higher education level (33% reported only primary or lower education compared with 41% among women in the lowest responsiveness category). Functional access was notably higher among women in the highest responsiveness category (87%) than women in the lowest category (55%). The regional distribution of women in responsiveness categories also varied, with a larger percentage of women from the Ashanti and Central regions in the highest responsiveness category and larger percentage of women from the Eastern, Greater Accra, Upper East and Upper West regions in the lowest responsiveness category. Women in the highest responsiveness category were more likely to have sought care in a private facility than women in the lowest category (22% vs 12%) and less likely to have sought care at government health centres (15% vs 30%). There were few differences in responsiveness categories by wealth quintile, urban/rural residence, reasons for care-seeking or person(s) for whom care was sought.

### Responsiveness and associations with patient outcomes

**Figure 2:**
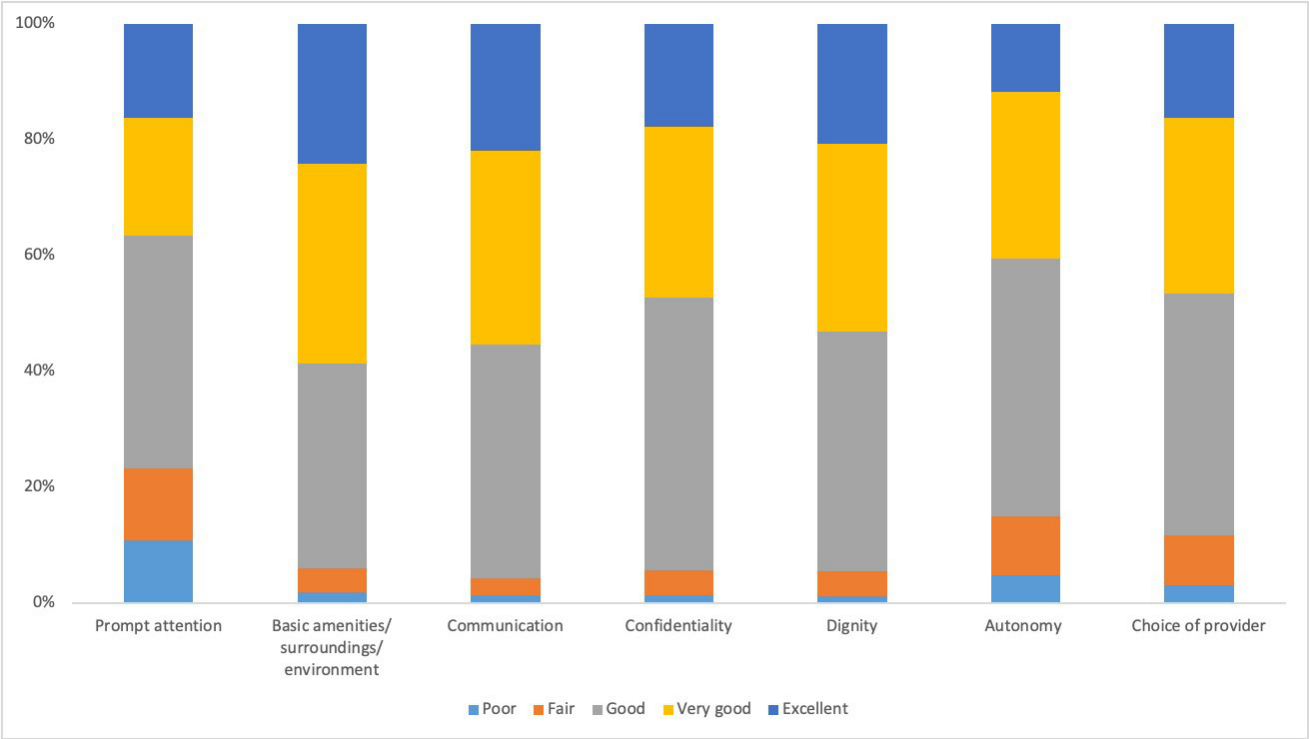
Responsiveness ratings by domain.

Among the seven responsiveness domains (figure 2), ratings were generally highest for quality of basic amenities (24% excellent), communication (22% excellent) and dignity (21% excellent). Ratings were lowest for autonomy (12% excellent, 15% fair or poor), choice of provider (16% excellent, 12% fair or poor) and prompt attention (16% excellent, 23% fair or poor). For all seven elements of responsiveness, respondent ratings were generally high, with only 4%–25% of respondents reporting ‘poor’ or ‘fair’ for any dimension. Data on the full distribution of patients’ rating of their experience during their most recent visit can be found in online supplementary table 2.

**Table 2:** Quality outcomes by responsiveness index quintiles

Patient ratings	Responsiveness index quintile					
	Q1 (N=549) n (%)	Q2 (N=321) n (%)	Q3 (N=392) n (%)	Q4 (N=280) n (%)	Q5 (N=404) n (%)	Total (N=1946) n (%)
Excellent last visit—met my health needs or helped me feel better	20 (3.6)	13 (3.9)	47 (12.1)	40 (14.3)	236 (58.4)	356 (18.3)
Excellent overall rating of quality of care at this facility	10 (1.9)	12 (3.8)	38 (9.6)	34 (12.1)	223 (55.0)	316 (16.3)
Highest recommendation of this facility to others	319 (58.1)	243 (75.7)	291 (74.3)	228 (81.6)	378 (93.4)	1459 (75.0)
Excellent self-rated health	46 (8.3)	26 (8.0)	62 (15.9)	76 (27.1)	136 (33.7)	346 (17.8)
Excellent self-rated mental health	31 (5.6)	19 (5.8)	54 (13.8)	56 (19.9)	128 (31.6)	287 (14.7)
Unmet need for family planning (only among those who sought care for self)*	89 (24.0)	60 (25.5)	55 (20.9)	39 (21.2)	71 (24.8)	315 (23.4)

Counts and percentages weighted to account for survey sampling design (unweighted n=2018).

Q1: lowest responsiveness quintile; Q5: highest responsiveness quintile.

*Unmet need for family planning was defined as fertile, sexually active women aged 15–49 who were not using contraception and did not wish to become pregnant for reasons of spacing (women who desire to postpone their next birth by a specified length of time) or limiting (women who desire no additional children).^28^ Counts and percentages for unmet need for family planning calculated within subset of those who sought care for themselves, so these have different denominators.

**Figure 3:**
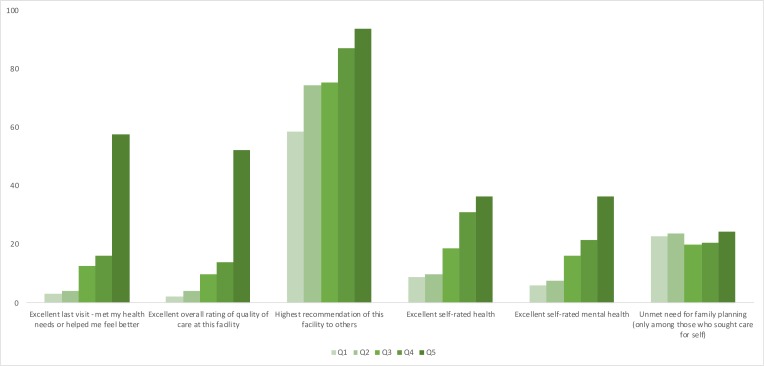
Quality outcomes by responsiveness index quintiles. Q1: lowest responsiveness quintile; Q5: highest responsiveness quintile.

Unadjusted results of the six outcomes are shown in table 2 and figure 3, broken down by responsiveness rating (for further details, see online supplementary table 3). Among patient satisfaction outcomes, which are specific to the respondent’s last visit, a strong, positive correlation was seen with higher ratings of responsiveness. Nearly one-fifth (18%) of respondents reported that their last visit was excellent in meeting their health needs, with notably higher reports among women in the highest responsiveness category than the lowest (58% excellent compared with 4%). Similarly, overall reports of excellent quality of care received at the last visit were 16%, with women in the highest responsiveness category being much more likely to report excellent quality (55%) compared with women in the lowest responsiveness category (2%). Overall reports of being extremely likely to recommend the facility to others were higher than other outcomes (75% overall), with women in the highest responsiveness quintile again more likely to report being very likely to recommend than women in the lowest category (93% compared with 58%).

**Table 3:** Relationships between responsiveness index quintiles and quality outcomes estimated using Poisson regression

	Responsiveness index quintile					
	Q1	Q2	Q3	Q4	Q5	P for trend
	PR (95% CI)	PR (95% CI)	PR (95% CI)	PR (95% CI)	PR (95% CI)	
Excellent at meeting health needs						
Unadjusted model 1	REF	1.1 (0.5 to 2.4)	3.4 (1.8 to 6.7)	4.0 (2.0 to 8.1)	16.4 (8.9 to 30.2)	<0.001
Adjusted model 2	REF	1.0 (0.5 to 2.2)	3.0 (1.6 to 5.5)	3.3 (1.7 to 6.5)	12.7 (7.0 to 22.9)	<0.001
Adjusted model 3	REF	1.0 (0.4 to 2.2)	2.9 (1.5 to 5.4)	3.1 (1.6 to 6.2)	13.0 (7.1 to 23.7)	<0.001
Excellent quality of care at this facility						
Unadjusted model 1	REF	2.1 (0.8 to 5.3)	5.3 (2.5 to 11.4)	6.6 (2.9 to 15.1)	29.9 (13.9 to 64.2)	<0.001
Adjusted model 2	REF	1.9 (0.8 to 4.8)	4.6 (2.2 to 9.8)	5.3 (2.3 to 12.3)	21.0 (9.9 to 44.6)	<0.001
Adjusted model 3	REF	1.9 (0.8 to 4.8)	4.6 (2.2 to 9.8)	5.0 (2.1 to 11.8)	20.8 (10.0 to 43.3)	<0.001
‘Very likely’ to recommend this facility to others						
Unadjusted model 1	REF	1.3 (1.1 to 1.5)	1.3 (1.1 to 1.5)	1.4 (1.1 to 1.7)	1.6 (1.4 to 1.9)	<0.001
Adjusted model 2	REF	1.2 (1.1 to 1.4)	1.2 (1.1 to 1.4)	1.3 (1.1 to 1.5)	1.4 (1.3 to 1.6)	<0.001
Adjusted model 3	REF	1.2 (1.1 to 1.3)	1.2 (1.1 to 1.4)	1.3 (1.1 to 1.5)	1.4 (1.2 to 1.6)	<0.001
Excellent self-rated health						
Unadjusted model 1	REF	1.0 (0.6 to 1.7)	2.0 (1.2 to 3.2)	3.4 (2.1 to 5.6)	4.2 (2.6 to 6.8)	<0.001
Adjusted model 2	REF	1.0 (0.6 to 1.7)	2.0 (1.2 to 3.3)	3.2 (2.0 to 5.2)	4.1 (2.3 to 7.1)	<0.001
Adjusted model 3	REF	1.0 (0.6 to 1.8)	2.0 (1.2 to 3.3)	3.1 (2.0 to 5.0)	4.0 (2.3 to 7.0)	<0.001
Excellent self-rated mental health						
Unadjusted model 1	REF	1.1 (0.6 to 2.2)	2.7 (1.3 to 5.4)	3.8 (1.9 to 7.8)	6.1 (3.0 to 12.1)	<0.001
Adjusted model 2	REF	1.1 (0.6 to 2.1)	2.6 (1.2 to 5.3)	3.2 (1.6 to 6.4)	5.1 (2.5 to 10.2)	<0.001
Adjusted model 3	REF	1.1 (0.6 to 2.1)	2.7 (1.3 to 5.5)	3.1 (1.6 to 6.2)	5.1 (2.6 to 9.9)	<0.001
Unmet need for family planning						
Unadjusted model 1	REF	1.2 (0.9 to 1.7)	0.9 (0.7 to 1.2)	1.1 (0.8 to 1.5)	1.1 (0.8 to 1.5)	0.845
Adjusted model 2	REF	1.2 (0.8 to 1.6)	0.9 (0.6 to 1.2)	1.0 (0.7 to 1.5)	1.0 (0.7 to 1.4)	0.697
Adjusted model 3	REF	1.2 (0.8 to 1.6)	0.9 (0.6 to 1.2)	1.0 (0.7 to 1.5)	1.0 (0.7 to 1.4)	0.676

Model 2 adjusted for age, wealth, education, urban/rural, ease of getting care tomorrow, same provider at each visit and region.

Model 3 adjusted for items in model 2, plus reason for seeking care, care for self or others, and facility type.

All models weighted to account for survey sampling design (unweighted n=2018).

Q1: lowest responsiveness quintile; Q5: highest responsiveness quintile.

PR, prevalence ratio; REF, Reference.

Among patient-reported outcomes, which were not specific to respondents’ last care-seeking experience, the association with responsiveness was strong but less uniform. Overall, 18% of respondents reported being in excellent overall health. Women in the highest responsiveness category more commonly reported excellent self-rated health (34%) than those in the lowest responsiveness quintile (8%). Self-reported mental health showed the same trend, with 15% reporting excellent mental health overall and reports of excellent mental health higher among women in the highest compared with the lowest responsiveness category. In contrast, unmet need for family planning (23% overall) did not show a strong positive relationship with responsiveness.

Table 3 presents the unadjusted and adjusted PRs for the six outcomes and responsiveness quintile after adjustment for respondent and visit characteristics. Five of the six outcomes were significantly positively associated (p<0.001) with responsiveness category, including models that adjusted for only respondent characteristics and models that adjusted for both respondent and visit characteristics. Women in the highest responsiveness quintile were 13.0 times as likely to report that the visit was excellent at meeting their health needs (95% CI 7.1 to 23.7), 20.8 times as likely to report excellent quality of care (95% CI 10.0 to 43.3), and 1.4 times as likely to report being very likely to recommend the facility to others (95% CI 1.2 to 1.6) compared with women in the lowest responsiveness quintile. Additionally, compared with women in the lowest responsiveness quintile, women in the highest responsiveness quintile were 4.0 times as likely to report excellent self-rated health (95% CI 2.3 to 7.0) and 5.1 times as likely to report excellent self-rated mental health (95% CI 2.6 to 9.9). We found no evidence of an association between responsiveness quintile and unmet need for family planning.

## Discussion

As recent major reports on healthcare quality have recently shown, universal health coverage cannot improve the health of a population if the coverage provided is not of high quality. A positive patient experience is critical to achieving high-quality care, and can improve patient retention, treatment adherence and patient satisfaction with the health system.^1^ Our study uses a WHO framework for measuring patient experience—responsiveness of care—and shows that care that is responsive to patients is tightly linked with higher ratings of satisfaction and health. Among women of reproductive age in Ghana, we found that responsiveness of care was strongly associated with perceptions that care met health needs, overall perceived quality of care, likelihood of recommending the facility, and self-rated health and mental health. To our knowledge, this is the first study to examine the relationships between individual-level patient-reported responsiveness and a suite of patient-reported satisfaction and outcome measures in a nationally representative survey of women of reproductive age in an LMIC. These relationships are illustrated in figure 1.

Although it is challenging to define a clear causal pathway between responsiveness and self-rated health—given the plethora of health system and non-health-related factors that may impact this outcome—our finding that responsiveness is associated with self-rated health is consistent with a recent study among urban men and women in six Latin American and Caribbean countries which found that those with positive ‘overall patient-centered primary care experience’, the definition of which has significant overlap with responsiveness, were 1.6 times as likely to report excellent or very good self-rated health.^17^ We did not find evidence that unmet need for family planning was associated with responsiveness of care at the last visit. This finding is perhaps not surprising given that only 7% of our sample reported seeking care specifically for family planning services, and an earlier PMA2020 study conducted among similar facilities found relatively low service availability and readiness, including that fewer than 60% of family planning services (as defined by the standard of care in Ghanaian policy) were provided.^22^ Our results highlight that responsiveness must be thought of as necessary and complementary to other foundations of health systems, including access to care and availability of essential supplies and commodities, as well as strong technical quality and provider competence.

Overall ratings of responsiveness were generally high, although areas such as prompt attention, autonomy and provider choice were rated lower than other areas, perhaps indicating a need for particular focus from policymakers and implementers in Ghana. Our findings indicate that women reporting less responsive care tended to be younger, less educated and less likely to report having access to care if needed quickly. Similar to an earlier study among older adults (age 50 and over) in six middle-income countries by Geldsetzer *et al*, our results showed that seeking care in a private facility compared with a public facility was associated with higher reports of responsiveness.^9^ Notably, our results did not find an association between respondents’ wealth and responsiveness of care, a finding in line with results from Ghana in Geldsetzer *et al*, but in sharp contrast to findings from countries such as India and South Africa.^9^ We also documented notable variation in regional distributions by quintile of responsiveness, with women from Eastern, Greater Accra, Upper East, Upper West and Western regions more likely to report lower responsiveness of care. These regions are economically and culturally diverse from one another, suggesting that issues underlying non-responsive care are likely to be varied and that increased national attention to improving responsiveness should be coupled with efforts to contextualise improvement strategies to the local setting across regions. Further underscoring this need for contextualisation, results from Geldsetzer *et al* found that, among older adults in Ghana and in contrast to our findings, age, education, and urban or rural residence were not associated with differences in responsiveness.^9^ These differences may reflect that predictors of responsiveness vary between relatively young women of reproductive age in our study and older adults of both sexes in Geldsetzer *et al*.^9^ These differences highlight the need for deeper study at the subnational level and within subpopulations to ensure that interventions to improve responsiveness take into account the multiple relevant perspectives and are person-centred in design.

Of particular note in our study is the magnitude of estimates of association between higher health system responsiveness and better patient-reported satisfaction, particularly in the domains of ‘meeting health needs’ and ‘overall ratings’ of care. Even after multivariable adjustment, those in the highest quintile of responsiveness were 13 times and 20 times as likely, respectively, to report their satisfaction in these domains as excellent compared with respondents in the lowest quintile. The magnitude of these estimates can be partially explained by the low percentages of women reporting excellent care in the lowest quintile of responsiveness compared with the highest quintile. Estimates of coefficients with strong effects and small counts can lead to statistical concerns about separation and sparse data bias^23 24^; however, we were able to address these concerns by using Poisson regression with robust SE in our primary analysis, which produces estimates that are less prone to unusually high values than odds ratios when higher magnitudes of association are present. We also performed a sensitivity analysis using Firth^23^ bias correction and smoothing methods to account for sparse data and found that this penalisation did not change the high magnitude of the estimates (data not shown). This difference in underlying distribution of reported ratings may also explain why some outcomes—for instance, the highest likelihood of recommending the facility to others, which was much more evenly distributed across responsiveness categories—have less extreme estimates of association. Nevertheless, even for an outcome like the likelihood of recommending, which showed much less variation by responsiveness quintile, the association between the two was robust across sensitivity analyses for all models run.

The robustness of these findings reflects an important association between responsiveness and satisfaction of care. Our results provide valuable insights to policymakers and implementers that improving overall person-centredness will require addressing the full range of health system responsiveness from waiting time to facility cleanliness to respect. Because our study uses observational, cross-sectional survey data, we cannot definitively determine whether the observed relationship between responsiveness and satisfaction and self-rated health outcomes is causal in any direction. However, growing evidence from LMIC shows that inadequate healthcare utilisation is the cause of millions of avoidable deaths per year,^1^ and that poor patient experience and patient satisfaction are major drivers of low utilisation.^1 25^ Our findings are aligned with the larger evidence base, which, taken all together, indicates that a lack of responsiveness may directly contribute to avoidable morbidity and mortality. In other words, responsiveness—which has long been thought of as a ‘nice to have’ feature of health systems and care delivery—is in fact indispensable for achieving better health, not a luxury that is only relevant for high-income settings. Users of healthcare systems vote with their feet for higher experiential and technical quality and aim to avoid perceived lower quality care. Future efforts are needed to ensure that responsiveness is more routinely measured and that this information is fed back to national and local policymakers and health system managers to inform priority setting and improvement efforts.

In addition to the challenges presented by the cross-sectional survey design, our study had a few other limitations. First, the PMA2020 surveys are designed to be nationally representative of women of reproductive age (15–49), so these results may not be generalisable to populations outside of this demographic or beyond the national level. Second, the survey was limited to women who had sought care within 6 months prior to the interview and may therefore represent a sample of women who seek care more frequently. Women who have not recently sought care may experience different levels of responsiveness and quality of care, so caution should be taken in extrapolating our results to the entire female population of Ghana. In particular, it is possible that some women who did not seek care in the last 6 months avoided doing so in part because of prior experience of unresponsive and unsatisfactory care. If true, the levels of responsiveness we found may be higher than those experienced in the general population. Additionally, women who sought care for themselves may rate responsiveness differently from women who sought care for someone else; however, a sensitivity analysis only among women who sought care for themselves found no major differences in the magnitude or direction of associations (data not shown). Finally, Ghana is a country with a strong historical investment in primary health care, universal health coverage and the public health sector, and the relationships described here may differ in health systems with different characteristics.^26 27^

## Conclusion

Among Ghanaian women of reproductive age, responsiveness is highly associated with patient satisfaction and patient-reported outcomes including self-rated overall and mental health. The documented inequity in reported responsiveness across respondent characteristics and geographical regions highlights that an increased national focus on improving responsiveness—coupled with locally adapted and tailored improvement plans—is needed in Ghana. More broadly, our results support the emerging global conclusion that responsiveness is both an intrinsic and instrumental goal for health systems, and one that is indispensable for achieving quality and meeting the promise of universal health coverage. That our study is one of the first to examine individual reports of responsiveness of care and its association with satisfaction and selected patient-reported outcomes at the population level demonstrates the relative neglect this topic has historically received. Moving forward, it must become a priority for policymakers, researchers and implementers everywhere to more systematically and routinely measure responsiveness and act on the findings in order to build health systems that meet people’s needs and deliver better health for all.
