# Supplementary material for: Towards patient-centred care in Ghana: health system responsiveness, self-rated health and experiential quality in a nationally representative survey
Source: BMJ Open Qual. 2020 May 12;9(2):e000886. doi: 10.1136/bmjoq-2019-000886 (PMC7228562; doi:10.1136/bmjoq-2019-000886)
Supplement: Supplementary data [file bmjoq-2019-000886supp002.pdf]

Supplemental Table 1: Demographic characteristics of women who sought care in the last six months and those who did not

	Sought care in the past 6 months (n=1,946)		Did not seek care in the past 6 months (n=2,293)	
	No.	%	No.	%
Age				
15 - 24	550	28.3	929	40.5
25 - 34	738	37.9	621	27.1
35 - 49	658	33.8	744	32.4
Primary or lower schooling	677	34.8	972	42.4
Income				
Bottom 2 wealth quintiles	774	39.8	796	34.7
Neighborhood				
Urban	971	49.9	1166	50.9
Rural	975	50.1	1127	49.1
Region of residence				
Ashanti	400	20.6	391	17.1
Brong Ahafo	110	5.7	117	5.1
Central	178	9.1	253	11
Eastern	229	11.8	222	9.7
Greater Accra	291	14.9	553	24.1
Northern	202	10.4	216	9.4
Upper East	133	6.8	103	4.5
Upper West	72	3.7	70	3.1
Volta	123	6.3	181	7.9
Western	207	10.7	188	8.2
Very easy to get care if needed tomorrow	1364	71.0	1492	65.1
Always see same provider	438	22.6	452	20.1

Supplemental Table 2: Respondent ratings across responsiveness categories

Responsiveness categories	Count	Proportion
**Rate waiting time**		
Poor	212	0.11
Fair	238	0.12
Good	781	0.40
Very good	400	0.20
Excellent	316	0.16
**Rate cleanliness**		
Poor	36	0.02
Fair	83	0.04
Good	688	0.35
Very good	671	0.34
Excellent	468	0.24
**Understand information**		
Poor	25	0.01
Fair	57	0.03
Good	787	0.40
Very good	651	0.33
Excellent	426	0.22
**Can talk privately**		
Poor	26	0.01
Fair	85	0.04
Good	916	0.47
Very good	576	0.30
Excellent	344	0.18
**Rate level of respect**		
Poor	25	0.01
Fair	83	0.04
Good	804	0.41
Very good	633	0.32
Excellent	402	0.21
**Involved in decisions**		
Poor	93	0.05
Fair	198	0.10
Good	865	0.44
Very good	560	0.29
Excellent	230	0.12

**Easy to see a provider**		
Poor	61	0.03
Fair	166	0.09
Good	813	0.42
Very good	590	0.30
Excellent	316	0.16

All proportions weighted to account for complex survey design

Supplemental Table 3. Quality of care ratings across responsiveness quantiles

	Responsiveness Index Quantile											
	Q1 (N = 549)		Q2 (N = 321)		Q3 (N = 392)		Q4 (N = 280)		Q5 (N = 404)		Total (N = 1946)	
	No.	%	No.	%	No.	%	No.	%	No.	%	No.	%
**Overall rating of care received**												
Poor	14	2.6	0	0	1	0.2	1	0.3	0	0	16	0.8
Fair	48	8.7	8	2.6	7	1.8	0	0.2	3	0.8	67	3.4
Good	378	68.7	191	59.5	124	31.6	71	25.3	29	7.1	792	40.7
Very good	90	16.4	109	33.9	213	54.3	168	59.9	137	33.8	716	36.8
Excellent	20	3.6	13	3.9	47	12.1	40	14.3	236	58.4	356	18.3
Total	549	100	321	100	392	100	280	100	404	100	1946	100
**Rate the quality of care at this facility**												
Poor	8	1.5	2	0.7	2	0.5	0	0.0	2	0.4	14	0.7
Fair	43	7.9	5	1.4	3	0.9	3	1.1	2	0.6	57	2.9
Good	382	69.6	182	56.6	133	34.0	50	17.8	33	8.2	780	40.1
Very good	105	19.1	120	37.5	215	55.0	193	69.0	145	35.7	779	40.0
Excellent	10	1.9	12	3.8	38	9.6	34	12.1	223	55.0	316	16.3
Total	549	100	321	100	392	100	280	100	404	100	1946	100
**Likelihood of recommending care at this facility to others**												
Very unlikely	31	5.7	8	2.4	7	1.8	2	0.6	3	0.7	51	2.6
Somewhat unlikely	15	2.8	4	1.1	6	1.5	2	0.7	3	0.8	30	1.5
Somewhat likely	184	33.5	67	20.8	88	22.5	48	17.1	21	5.2	407	20.9
Very likely	319	58.1	243	75.7	291	74.3	228	81.6	378	93.4	1459	75.0
Total	549	100	321	100	392	100	280	100	404	100	1946	100
**Overall health rating**												
Poor	27	4.9	14	4.5	10	2.6	14	5.0	32	7.8	97	5.0
Fair	63	11.4	41	12.7	47	12.1	41	14.7	38	9.3	229	11.8
Good	301	54.8	137	42.7	114	29.1	53	19.0	85	21.1	690	35.5
Very good	113	20.5	103	32.1	158	40.3	96	34.2	114	28.2	583	30.0

Excellent	46	8.3	26	8.0	62	15.9	76	27.1	136	33.7	346	17.8
Total	549	100	321	100	392	100	280	100	404	100	1946	100
**Mental health rating**												
Poor	25	4.5	22	6.8	10	2.6	16	5.6	52	12.8	124	6.4
Fair	92	16.8	45	13.9	52	13.3	36	13.0	37	9.1	262	13.5
Good	297	54.0	160	49.8	136	34.8	63	22.6	71	17.6	727	37.4
Very good	105	19.1	76	23.7	139	35.5	109	38.9	117	28.9	546	28.0
Excellent	31	5.6	19	5.8	54	13.8	56	19.9	128	31.6	287	14.7
Total	549	100	321	100	392	100	280	100	404	100	1946	100
**Unmet need for family planning**												
No	281	76.0	176	74.5	210	79.1	144	78.8	216	75.2	1027	76.6
Yes	89	24.0	60	25.5	55	20.9	39	21.2	71	24.8	315	23.4
Total	370	100	236	100	265	100	183	100	288	100	1341	100
